# Patient-recorded outcome to assess therapeutic efficacy in protoporphyria-induced dermal phototoxicity: a proposal

**DOI:** 10.1186/1477-7525-8-60

**Published:** 2010-06-21

**Authors:** Elisabeth I Minder, Xiaoye Schneider-Yin, Christoph E Minder

**Affiliations:** 1 Stadtspital Triemli, Zentrallabor, Birmensdorferstrasse 497, CH-8063 Zürich, Switzerland; 2 Horten Centre for Patient Oriented Research and Knowledge Transfer, University Hospital of Zürich, Zürich

## Abstract

**Background:**

Protoporphyria (PP) resulting from two rare, inherited diseases of heme biosynthesis leads to dermal phototoxicity by accumulation of the heme precursor protoporphyrin IX. No standardized tools to quantify the degree of PP-related phototoxicity and its change by medical intervention have been published.

**Methods:**

Results from a questionnaire completed by 17 affected individuals were used to determine the relative importance of two main components of PP-related phototoxicity, skin pain and sunlight exposure time, with respect to the effectiveness of any particular medical treatment.

**Results:**

Inter-rater reliability was 0.71 (n = 490), repeated estimates by four identical individuals showed high reproducibility (Slope = 1, intercept = 0, n = 136, Passing-Bablock).

Six different models were developed, three of them showed good correlation with effectiveness estimates. Data from an unpublished trial indicated that the model with highest potential of responsiveness was the so called "**E**xposure **t**imes [multiplied by] **F**reedom from **P**ain" (ETFP). The minimal clinically important difference (MID) was 15 (10.2-20.4) ETFP scores, representing 28% of the standard deviation of the clinical trial data and 2.9% of its total range.

**Conclusions:**

Among the six models proposed to assess the effectiveness of therapeutic interventions in PP the ETFP model demonstrates the highest sensitivity using the existing data from a clinical trial of afamelanotide in PP. The results of this study have provided sufficient validation of the ETFP model that is likely to prove useful in future clinical trials.

## Background

Erythropoietic protoporphyria (EPP, OMIM 177000), a rare inherited disease of heme biosynthesis, is due to mutations of the enzyme ferrochelatase that catalyzes the ultimate step in heme biosynthesis, the insertion of iron into protoporphyrin IX to form heme [1,2]. Recently, a new disease entity, X-linked protoporphyria (XLDPT; OMIM 300752), which is caused by an over-activity of aminolevulinic acid synthase 2 due to specific mutations in its C-terminal region has been described [2,3]. Protoporphyria (PP) refers to both EPP and XLDPT in this article. The main symptom, dermal phototoxicity, is identical in both diseases, as they both lead to an accumulation of the ferrochelatase substrate, photosensitizing protoporphyrin IX. The accumulated protoporphyrin is composed of two fractions, zinc-protoporphyrin and (metal-)free protoporphyrin. Patients with XLDPT show a higher proportion of zinc-protoporphyrin than those with EPP. As zinc-protoporphyrin does not induce phototoxicity [4,5], patients with XLDPT may exhibit less phototoxicity than classical PP patients at the same level of total erythrocytic protoporphyrin. Due to the hydrophobicity of protoporphyrin, excess protoporphyrin is eliminated only by the biliary route. In about 1-4% of PP patients a protoporphyrin-induced liver failure develops, heralded by increasing erythrocytic protoporphyrin levels and concomitant increment in phototoxicity [6].

Light-induced phototoxic reactions in PP are of variable severity: Immediately or within a few minutes of sunlight-exposure, PP-patients feel stinging pain in sun-exposed skin that disappears upon termination of light-exposure. On prolonged exposure, erythema, edema and skin lesions may develop and an incapacitating pain may occur. The pain cannot be alleviated by pain killers such as acetaminophen or salicylic acid derivatives. Even non-steroidal-anti-rheumatics are ineffective. The severity of phenotype is related to erythrocytic or plasma protoporphyrin levels [7].

Our recent systematic literature review on treatment options of dermal phototoxicity in protoporphyria (PP) showed that available publications are of insufficient quality to prove efficacy of any treatments that have been proposed to date [8]. A major problem revealed by this study was the lack of a standardized efficacy assessment. Consequently, diverse assessment techniques were applied among different studies which made it difficult to compare their results.

Dermal phototoxicity in PP is largely a subjective perception because initial symptoms are rarely accompanied by physical signs. An optimal measure of subjective symptoms is the recording of patients' experience [9-11]. Tools for such purposes have been named 'patient recorded outcome' (PRO) determinations. They are frequently, but not exclusively, designed in the form of questionnaires. Recently published guidelines and articles have defined the necessary scientific quality of PRO's[12-14]. Generalized and standardized tools such as SF36 with documentation of these requirements are available. But often they do not target specific symptoms of a particular disease resulting in a low sensitivity in detecting important treatment-induced changes [15]. Therefore, in many instances disease-specific PRO's have been applied [16]. The most frequently used PRO in dermatological diseases is the 'dermatological quality of life index' (DLQI). This well documented tool [17] has also been applied in measuring the life quality in PP[7], but it never has been used to evaluate the effect of treatment during the acute phase of PP.

The scientific value of a disease-specific PRO is dependent on the following criteria: Rationale for choosing selected endpoints, documentation of psychometric characteristics (content and construct validity, reliability and responsiveness) and interpretation guidelines including minimal important difference [18]. Evaluations of some of these criteria require the availability of a documented effective treatment, which is not available in PP [8]. Here, we propose a PRO instrument for the therapeutic evaluation of dermal phototoxicity in PP.

## Methods

### Data source

Three different information sources for development and verification of various models were used: (1) a systematic review on treatment options of dermal phototoxicity in erythropoietic protoporphyria rendered information on possible items reflecting the severity of phototoxicity (2) a questionnaire described below for the construction of an optimized model (3) unpublished data from a trial of afamelanotide in PP (Trial No ACTRN12607000261415) for checking additional aspects of the model. This work was conducted according to the Declaration of Helsinki and has been approved by the institutional and cantonal ethics review board (Ethik-Kommission der beiden Zürcher Stadtspitäler, STZ 07/07).

### Rational for choosing endpoints

As stated in the Introduction, skin pain of variable intensity is the main symptom of PP-related phototoxicity. Conditioned by their immediate pain reaction upon sunlight-exposure and by their life-long experience of the incapacitating pain from severe phototoxic reactions, adult PP-patients are often able to anticipate the impending risk of phototoxicity depending on the actual weather. In case of presumed high risk of phototoxicity, patients tend to avoid sunlight exposure as much as possible. If sunlight-avoidance is strictly followed, patients no longer suffer from phototoxicity, but the disease markedly limits outdoor-activities and activities in rooms lit by direct sunlight and thus, it affects social and working capabilities of the patients.

A PRO to determine PP-related phototoxicity contains therefore two components: pain and sunlight exposure. A daily recording of both pain intensity and sunlight exposure time reflects the actual functioning of the patient. The two components, pain and sunlight exposure interact with each other, as patients suffering from pain will decrease their sunlight exposure and patients who extend their sunlight exposure will increase their risk of pain. A tool was therefore developed to include both components and was tested for its ability in documenting the effect of a medical treatment on acute disease activity in PP.

### Content validity

For content validity, patients and clinicians should be involved in identifying and confirming the content of measure. Here, we relied on the information obtained from a systematic review on therapeutic studies in PP and the tools used in these studies to assess effectiveness [8]: Both pain intensity and time of light tolerance were the efficacy measurements used with light tolerance being the preferred endpoint.

### Construction of a model

Construct validity refers to the degree to which the measure reflects what it is supposed to measure rather than something else. In the case of PP, the goal was to construct a model that enables a reliable quantitative measure of the construct 'PP-related dermal phototoxicity' for the purpose of determination of the effectiveness of medical interventions.

The model required the establishment of a relationship between the effectiveness of a particular medical intervention and the phototoxicity score. As outlined above, time of sunlight tolerance and pain intensity are the two main and interdependent factors in PP-related phototoxicity. Due to the subjective nature of phototoxicity, only PP-affected persons can define the relative importance of these two factors for their well-being. To quantify the relative weight of both factors, a questionnaire was developed and sent to 27 affected persons. An estimate of effectiveness of any particular medical treatment was requested if, after variable sunlight exposure, a specified pain intensity resulted. The proposed sunlight exposures were 15 min, 30 min, 1 hour, 3 hours, 6 hours, 10 hours and 12 hours. Under each of these exposure times, pain intensities of none, mild, moderate, severe and intolerable were separately listed (table 1). For each position, the effectiveness of any particular medical treatment was estimated on a scale of 0 and 100%. An environmental condition to which estimates apply the season and the daytime with highest phototoxic risk was defined.

**Table 1:** Questionnaire A: Please estimate the minimal effectiveness of a particular medical treatment for EPP in percent between 0 and 100.

Exposure	Pain reaction	% Effectiveness (between 0 and 100)
After 12 hours sunlight exposure:	1.1. You suffer from intolerable pain	

	1.2. You suffer from strong pain	

	1.3. You suffer from moderate pain	

	1.4. You suffer from mild pain	

	1.5. You don't suffer from pain	

		

After 10 hours sunlight exposure:	2.1. You suffer from intolerable pain	

	2.2. You suffer from strong pain	

....After 6, 3, 1 hour, 30 minutes...		

After 15 minutes sunlight exposure:	7.1. You suffer from intolerable pain	

	7.2. You suffer from strong pain	

	7.3. You suffer from moderate pain	

	7.4. You suffer from mild pain	

	7.5. You don't suffer from pain	

During summer time (season of the highest risk) you expose yourself to the sun and afterwards you have a reaction specified below

Only 7 questionnaires were correctly filled in and participants mentioned that the questionnaire was difficult to understand. Therefore 12 of the initially addressed 27 individuals received additional explanations by the interviewer (EIM) during a regular a medical visit. Care was taken not to influence the estimates by highlighting the intended context only. Ultimately, 17 PP-patients felt sufficiently at their ease to answer each of the 35 lines in the questionnaire, resulting in a total of 490 estimates.

Using an 11-point Lickert scale as a reference, pain intensities were converted into pain scores so that no pain equaled to 0, mild to 2, moderate to 5, severe to 8 and intolerable to 10 scores, respectively. Sunlight exposure times were converted in 15-minute blocks. Inter-rater reliability was assayed by the method of Ebel http://www.med-ed-online.org/rating/reliability.html, accessed 3^rd ^Aug. 2009).

### Reliability

Reliability refers to the consistency with which an instrument measures a given construct [12] and determines to what extent an error is present in the instrument [19]. It has two components: the internal consistency measured by Cronbach's alpha and test-and-retest reliability or repeatability [20]. The determination of Cronbach's alpha requires a multi-item assessment. The patient ratings on these items are statistically related to each other so as to estimate the underlying construct, and Cronbach's alpha is a measure of the statistical relatedness of the items [21]. Cronbach's alpha was calculated from the data of an unpublished phase III trial of afamelanotide in PP.

Repeatability requires testing and retesting of stable patients. PP-related phototoxicity occurs in separate attacks; therefore patients do not exhibit stable symptoms. Hence repeatability assessment was replaced by analyzing the reproducibility of effectiveness estimates from the same individuals 4 months after the initial inquiry. The questions were rephrased with "pain intensities" as the main attribute (first column in table 2) as opposed to "sunlight exposure time" in the first questionnaire (table 1). For this second assessment, no verbal explanations were given.

**Table 2:** Questionnaire B: Please estimate the minimal effectiveness of a hypothetical medical treatment for EPP in percent between 0 and 100.

Pain reaction	Exposure	% Effectiveness (between 0 and 100)
You suffer from intolerable pain:	1.1. After 12 hours of sunlight exposure	

	1.2. After 10 hours of sunlight exposure	

	1.1. After 6 hours of sunlight exposure	

	1.2. After 3 hours of sunlight exposure	

	1.1. After 1 hour of sunlight exposure	

	1.2. After 30 minutes of sunlight exposure	

	1.1. After 30 minutes of sunlight exposure	

		

You suffer from strong pain:	2.1. After 12 hours of sunlight exposure	

	2.2. After 10 hours of sunlight exposure	

....you suffer from moderate pain; you suffer from mild pain...		

You don't suffer from pain:	5.1. After 12 hours of sunlight exposure	

	5.2. After 10 hours of sunlight exposure	

	5.3. After 6 hours of sunlight exposure	

	5.4. After 3 hours of sunlight exposure	

	5.5. After 1 hour of sunlight exposure	

	5.6 After 30 minutes of sunlight exposure	

	5.7 After 15 minutes of sunlight exposure	

During summer time (season of the highest risk) you expose yourself to the sun and afterwards you have a reaction specified below

### Responsiveness, Minimal important difference (MID)

Responsiveness is determined by evaluating the relationship between changes in clinical or patient-based endpoints and changes in the score [22]. The use of an unresponsive instrument will result in a failure to demonstrate statistical and clinical significance regardless of the true treatment effect [12,23]. The MID has been defined as the smallest difference in scores of a PRO measure that is perceived by patients as beneficial or harmful, and that could lead a clinician to consider a change in treatment [24]. Thus, a MID represents not only a statistically but also a clinically significant difference. MID ensures that the observed difference between treatment groups exceeds what one might expect based upon measurement error alone. Distribution-based methods for the assessment of MID rely on baseline variability of baseline scores. As mentioned above, phototoxicity has a high degree of variability due to its episodic character. Therefore distribution based methods were considered inappropriate. Instead, two anchors were used: A Lickert type pain scale and a global rating. The MID of a 7-point Likert-type pain scale is 0.5 points difference [25]. As the pain score used in this study has 11 points, the MID was converted to 0.5 *(11/7) or 0.8. The second anchor could be considered as a type of 'global rating of change'. The frequency distributions of the effectiveness estimates in the questionnaires were analyzed. Patients had to make an estimate on a 101-point effectiveness scale, which can be considered as a continuous scale. If specific values cumulate, the interval of these values was assumed to reflect the minimal difference in change that patients consider discernible.

### Statistical tools

Statistical tests were performed by Analyse- it- for- excel, version 2.11, by Vassar Stats http://faculty.vassar.edu/lowry/VassarStats.html, accessed July-August 2009) or by inter-rater calculator according to Ebel RL [26]http://www.med-ed-online.org/rating/reliability.html accessed Aug 2009). Stata version 10 was used.

## Results

### Construction of a model based on questionnaires

#### Inter-rater reliability of effectiveness estimates

**Figure 1:**
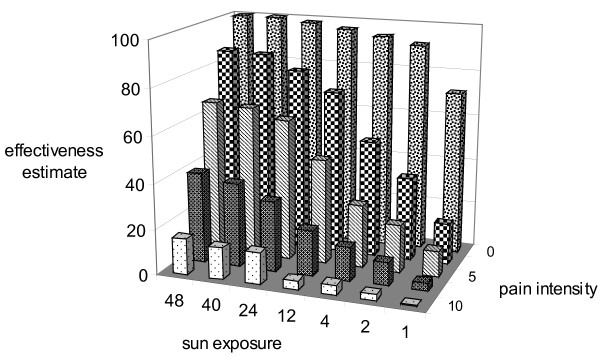
**The effect of sunlight exposure time and pain on effectiveness estimate**. The means of 490 estimates of effectiveness are plotted against both pain levels and sunlight exposure time. The pain scores are: 0 = no pain, 2 = mild pain, 5 = moderate pain, 8 = strong pain, 10 = intolerable pain. Exposure times are expressed as "multiples of 15 minutes", e.g. 1 = 15 min, 10 = 2.5 hours, 48 = 12 hours etc. The effectiveness ratings are in percent between 0 and 100. It is evident, that pain has a higher influence on the effectiveness rating than sunlight exposure time.

Inter-rater reliability was calculated among 17 individuals who filled out the 35-line questionnaire. The reliability for a score was 0.71 based on one rater. Both, pain and exposure time independently influenced the effectiveness estimates (Fig 1). As expected, the less the pain and the longer the exposure time, the higher were the effectiveness estimates.

#### Repeatability of effectiveness estimates

**Figure 2:**
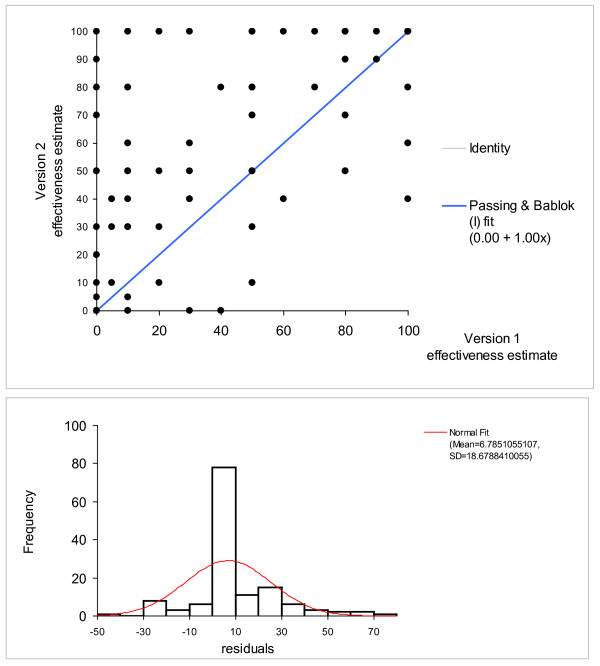
**Repeatability, scatter blot and histogram of residuals according to Passing-Bablok, n = 490 **[34]. The variability of the estimates may be overestimated in scatter blot. The histogram of residual reveals that many estimates lie close to zero. Consistently, the slope of the diagram is one, the intercept zero.

Four out of the 17 patients provided a second estimate. The repeated estimates showed a good repeatability (Spearman's rs = 0.82) and the Passing-Bablok test showed a high degree of reproducibility (intercept = 0, slope = 1; Fig 2), the 95% confidence intervals overlap with the regression line in the graph. This finding confirms the good rater reliability.

#### Contribution of phototoxicity components to effectiveness estimates

Both phototoxicity components, pain intensity and sunlight exposure time, correlated with the effectiveness estimate of patients (Spearman's rs -0.73 for pain intensity and 0.36 for sunlight exposure time). The variable pain intensity measured on a Lickert type scale was called either "pain" or "pain score". Sunlight exposure time was named either "exposure time" or "exposure". The effectiveness estimate was used as the independent variable with pain intensity or sunlight exposure time as the dependent variables. As expected, pain correlated inversely and sunlight exposure time correlated directly with effectiveness (data not shown). The different directions of correlation required to first invert the direction of one of the two components so that both components were in the same direction, and then to combine the two components into a single score i.e. either sum or product. The following conversions were performed: 'freedom from pain' was defined as 10 minus pain score, 'sun avoidance' was defined as 13 hours minus sun exposure time in 15-min blocks. The scale of 'freedom from pain' ranged from minimum 0 to maximum 10 scores, where zero meant intolerable pain and 10 means no pain; that of 'sunlight avoidance' ranged maximum 52 to minimum 0, where 52 meant no sunlight exposure and 0 means 13 hours of sunlight exposure within a day.

Five different models each comprised of two components, pain intensity and sunlight exposure, were tested for correlation with estimated efficacy by both linear regression analysis and the Spearman's correlation (table 3). In the formulas below, 'P' represents variable pain (intensity or score), 'E' represents variable (sunlight) exposure time.

**Table 3:** Correlation of the different models to the effectiveness estimates.

Model	Spearman's rs (95%confidence interval)	R^2^-linear regression
ETFP	0.74 (0.69 to 0.77)	0.40

PTSA	-0.77 (-0.80 to -0.73)	0.50

PDE	-0.78 (-0.81 to -0.74)	0.27

SE&FP	0.54 (0.47 to 0.60)	0.22

SA&P	-0.54 (-0.60 to -0.47)	0.22

E&P&EP	0.81 (0.77 to 0.84)	0.65

pain	-0.73 (-0.77 to -0.69)	0.54

sunlight exposure	0.36 (0.28 to 0.44)	0.10

**Figure 3:**
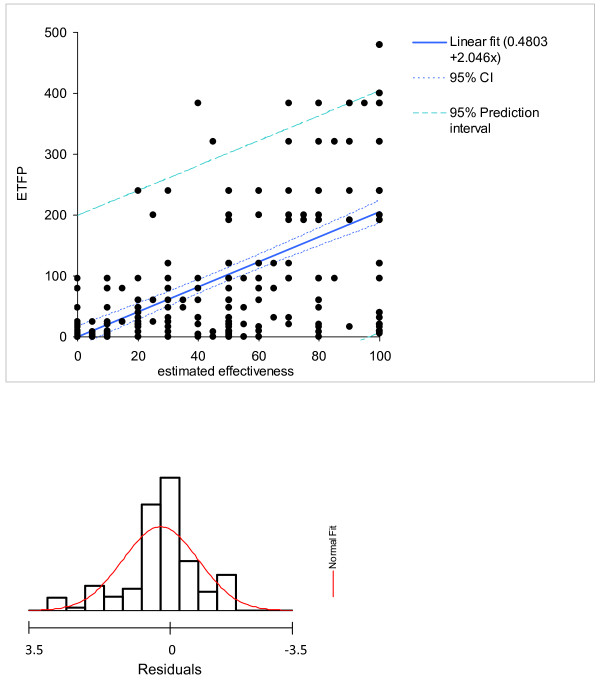
**The ETFP model**. ETFP is directly related to the effectiveness estimates. The scatter blot displays 490 estimates; the number of visible observations is reduced by superposition of those observations, as shown by the histogram of residuals.

• *Model 1: *P/E = **P**ain intensity **D**ivided by sun **E**xposure time (PDE).

• *Model 2: *E*(10 - P) = **E**xposure time **t**imes **F**reedom from **P**ain (ETFP, fig. 3).

• *Model 3: *P*(52 - E) = **P**ain **t**imes **S**un **A**voidance (PTSA).

• *Model 4: *E+ (10 - P) = **S**um of **E**xposure time **plus F**reedom of **P**ain (SE&FP).

• *Model 5: *(52-E) + P = **S**um of **S**un **A**voidance **plus P**ain (SA&P).

All five models were significantly correlated with estimated efficacy, p < 0.0001. PDE, SE&FP and SA&P showed less correlation with effectiveness estimates than PTSA and ETFP (table 3). Whereas the models with either multiplication or division were independent from the relative scales of the items (PDE, ETFP and PTSA), the models using sum of items were not order-invariant (SE&FP, SA&P).

Models 2 to 5 can be expressed by the same formula M = a +b*sunlight exposure + c*pain score +d*sunlight exposition * pain score. For example, model 2 (ETFP) is represented by factors a = 0, b = 10, c = 0 and d = -1. Based on the questionnaire data, the values of a, b, c and d and their standard errors were estimated by linear regression as follows: a = 69.5 SE 2.6, b = 0.854 SE 0.103, c = -7.55 SE 0.43, d = -0.0244 SE 0.0165; r^2 ^= 0.648. This resulted in

**Figure 4:**
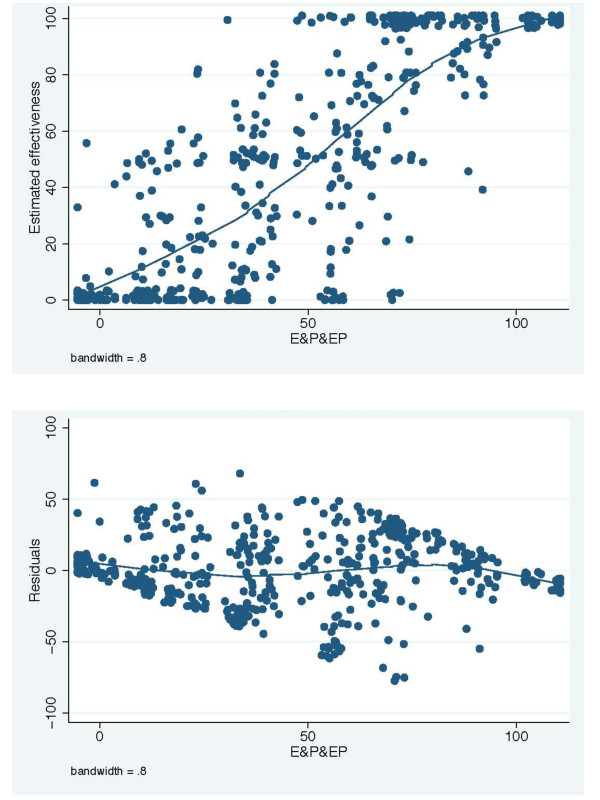
**The E&P&EP model fitted to efficacy estimates (A) and a graph of residuals versus linear prediction (B)**.

• *Model 6: *E&P&EP = 69.5 + 0.854 *Exposure -7.55*Pain score -0.0244 *Exposure * **P**ain score (fig. 4)

#### The meaning of PTSA, ETFP and E&P&EP

**Figure 5:**
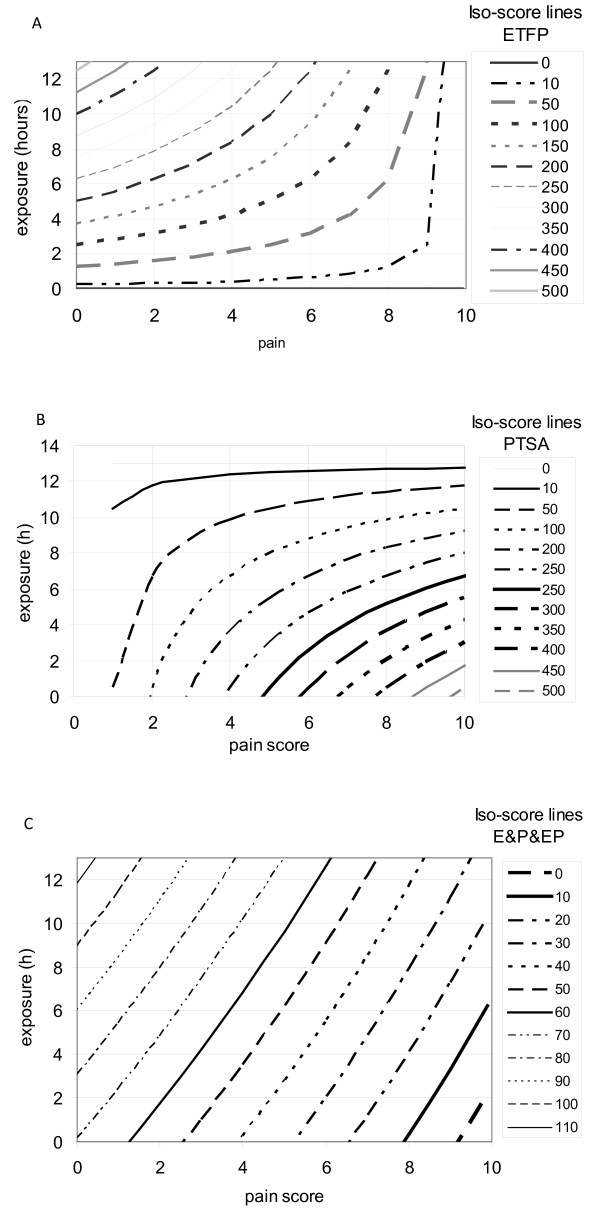
**ETFP (A), PTSA (B) and E&P&EP(C) in relation to sun exposure time and pain intensity**. The lines represent identical values (iso-score lines); the exact values are given in the legend on the right side. Examples given in the text serve to improve the readability. ETFP has a better discriminatory power at long exposure times, whereas PTSA has better discriminatory power at short exposure times. E&P&EP has a nearly identical discriminatory power over the whole range of exposure and pain scores.

Scores derived from multiplications are less intuitively understandable than those derived from additions. To illustrate these abstract tools, the relations pain and exposure time in ETFP, PTSA or E&P&EP are plotted in fig. 5A, B and 5C separately. The lines displayed represent identical scores, called iso-scores, for ETFP, PTSA or E&P&EP, respectively. These figures show that a patient exposed to sunlight for 4 hours and feeling a pain intensity of 4 has the same ETFP score of 100, as one exposed for 6 hours and feeling a pain intensity of 6. However, if after 6 hours of exposure, the patient felt only a pain intensity of 2, the ETFP score would be approximately 200. On the other hand, a patient exposed for 6 hours and feeling a pain intensity of 2 has roughly the same PTSA score (50) as one exposed for 10 hours and feeling a pain score of 4. In Model 6 (E&P&EP), nearly straight lines represent identical scores illustrating that the product E*P has only a low effect on the model.

These figures show that the power of discrimination in ETFP is high at long exposure times, as illustrated by the high number of iso-score lines crossing the horizontal lines, e.g. the line indicating 10-hour exposure time is crossed by ETFP iso-score lines from 50 to 400, depending on the pain intensity. In contrast, PTSA has high discriminating power at shorter exposure times. At a maximum exposure time of 13 hours, PTSA is independent of pain intensity and at long exposure times, different pain intensities influence the score only marginally. As it is unlikely that a patient suffers from severe pain after short exposure time, it is assumed that the high discriminatory power at low exposure times is of a less practical importance than that at long exposure times. This finding implies that ETFP is likely to be more responsive to treatment effects than PTSA. E&P&EP and ETFP are similar models except that high pain intensity influences and depresses ETFP scores more than it does E&P&EP.

Overlay of data from the afamelanotide trial with the different iso-score line plots showed that most data clustered at the origin of coordinate axes (data not shown). These data are therefore not informative with respect to drug efficacy in the clinical trial. Informative data are those that feature either long exposure and low pain levels or high pain intensities after moderate to long exposures. As ETFP is highly discriminatory for both areas, ETFP could have some advantage compared to E&P&EP. However, only the data that are obtained from clinical trials on effective substances will enable the responsiveness between the two models to be compared.

#### Estimation of the minimal important difference of ETFP

**Figure 6:**
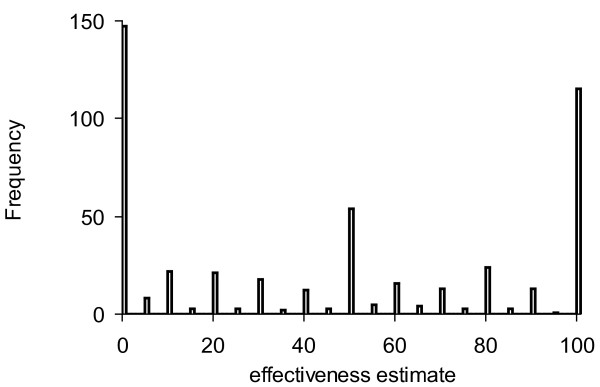
**Frequency histogram of the effectiveness estimates**. The abscissa displays the 101-point estimation scale (0% to 100%), the ordinate the number of estimate per scale point. Ten percent and multiples thereof appear more frequently than 5% estimates. Estimates in between the 5% intervals are missing.

One anchor to define the MID was the pain score which correlated linearly with the ETFP score in the data derived from the questionnaires. A MID of 0.8 on the 11-point Lickert scale and the correlation between ETFP and pain were used for the estimation (ETFP = -18.71 × painscore+187.1; r^2 ^= 0.28). A MID of 15.0 ETFP-scores resulted. The second anchor derived from the frequency distribution of the effectiveness estimates of the patients. The patients chose between 0 and 100% on a 101-scale. However, they preferred certain values, as illustrated by a histogram of the frequency of levels chosen (fig 6). The steps used were 10% or multiples thereof and 5% at a lower frequency. Other values were rarely used. Apparently, the patients intuitively felt that they will not perceive a change below five to ten percent of effectiveness. The range 5-10% effectiveness was projected on the regression line correlating effectiveness to ETFP (fig. 3; ETFP = 2.046[effectiveness estimate]). The resulting MID was 20.5 ETFP scores for the 5% interval and 10.2 for the 10% interval. The MID scores for ETFP estimated by different anchors were comparable, implying an ETFP score of 5 to 10 can be used as MID. The analogous procedure performed for the model 6 (E&P&EP) resulted in a MID of 6.4 for the anchor pain and 15.4 for the 10% and 7.7 for the 5% effectiveness anchors.

## Discussion

A standardized quantitative assessment on PP-related dermal phototoxicity to analyze effects of therapeutic interventions has not previously been published. Moreover, experts in the field considered determination of efficacy in PP difficult [27,28].

In this work, models for quantitatively assessing PP-related dermal phototoxicity were proposed. It was not our intention to develop a measure of phototoxicity per se e.g. a score enabling the comparison of phototoxicity intensity among different skin diseases.

The models are based on two components, sunlight exposure times and pain intensity scores. These components were chosen according to a systematic, comprehensive literature review on therapeutic studies in PP [8]. We assume that daily recording of these components by diaries and/or electronic means are necessary for the generation of reliable data.

Obviously, other variables such as sunlight intensity and sunlight color influenced by geographical latitude, season and weather, individual pain sensitivity, protoporphyrin concentration in blood, social and psychological factors may also influence phototoxicity and therefore inevitably introduce measurement errors to simple models that are limited to sunlight exposure and pain. The evaluation of the proposed models faced several problems: (1) Whereas most PRO's are related to stable disease situations, PP is characterized by attacks; (2) PRO's are validated by comparison to another quantitative standardized measure, which has not been published for PP; (3) The responsiveness of PRO is validated by an effective treatment, which is also not available in PP.

To overcome such limitations, we started from PP-patients' expectation of the effectiveness of any medical treatment, and calculated quantitative correlation factors of the different models with the patients' expectations. The three models 'E&P&EP', 'PTSA' and 'ETFP' were found to best represent these expectations. Distribution of iso-score lines made plausible that ETFP may show highest responsiveness.

Internal consistency, one component of model reliability, tests for correlations among different items that constitute a PRO. Cronbach's alpha, the measure of internal consistency, should be above 0.7 to support acceptable reliability [29]. Cronbach's alpha is -according to our knowledge - defined for summation scores only. Cronbach's alpha of the above mentioned unpublished trial data was negative (-0.118) when determined from pain, sunlight exposure and the summation model 'SE&FP'. The components of our models, sunlight exposure time and pain score, are complementary rather than highly correlated information. Therefore, models composed of these two components represent a multiple cause indicator model rather than a multiple effect indicator [21], as the items of this model are not interchangeable and thus have a weak correlation. Consequently, they represent more than one dimension, which explains the negative Cronbach's alpha.

The proposed model showed a good inter-rater reliability of 0.71, well above the acceptance level of 0.6, indicating that the patients have very comparable expectations towards effectiveness of a medical treatment. This finding was surprising, because the disease severity as measured by the DLQI varied considerably among PP-patients [7]. DLQI measuring quality of life rather than PP-related phototoxicity, is not directly comparable with this model and the DLQI data derived from a much larger PP-patient sample than in the afamelanotide trial. It remains to be examined whether DLQI could serve as a complementary measure to dermal phototoxicity in clinical trials on PP.

The distribution of iso-score lines suggested a higher responsiveness for the 'ETFP' than for the E&P&EP model. MID estimated by two different anchors were 15 (10-20) ETFP scores and 6.4 (7.7-15.4) E&P&EP scores. A comparison of these values to the afamelanotide trial data will illustrate the potential responsiveness in a clinical trial. As ETFP-scores exhibited a standard deviation of 53 and a range from 0 to 520 in the afamelanotide trial, the MID_ETFP _equals 28% of the standard deviation of the trial data, and 2.9% of the total range. The MID_E&P&EP _was equal to 96% of the standard deviation and to 5.6% of total range. These values imply that the sensitivity for assessment of changes in dermal phototoxicity is higher for the ETFP model than for the E&P&EP model. The ETFP model may therefore serve in the future as a tool to evaluate efficacy of therapeutic interventions in PP, such as treatment by narrow band UV[30,31], application of alpha MSH analogues [32,33] or one of the other numerous treatments proposed in PP [8].

## Conclusion

Among the six models proposed to assess the effectiveness of therapeutic interventions in PP the ETFP model demonstrates the highest sensitivity using the existing data from a clinical trial of afamelanotide in PP. The results of this study have provided sufficient validation of the ETFP model that is likely to prove useful in future clinical trials.

## Abbreviations

EPP: erythropoietic protoporphyria; XLDPT: X-linked dominant protoporphyria; PP: Protoporphyria; PRO: Patient-reported outcome; DLQI: dermatological life quality index; MID: minimal important difference; P/E: **P**ain intensity **D**ivided by sun **E**xposure time (PDE), Model 1; E*(10 - P): **E**xposure time **t**imes **F**reedom from **P**ain (ETFP), Model 2; P*(52 - E): **P**ain **t**imes **S**un **A**voidance (PTSA), Model 3; E+ (10 - P): **S**um of **E**xposure time **plus F**reedom of **P**ain (SE&FP), Model 4; (52-E) + P: **S**um of **S**un **A**voidance **plus P**ain (SA&P), Model 5; E&P&EP: 69.5 + 0.854 *Exposure -7.55*Pain score -0.0244 *Exposure * **P**ain score, Model 6.

## Competing interests

The authors declare that they have no competing interests.

## Authors' contributions

EIM developed the study design, contributed to the acquisition of data, their analysis and interpretation and wrote the manuscript, CEM was involved in the study design, XSY and CEM assisted in the analysis and interpretation of data and critically reviewed and revised the manuscript. All authors have read and approved the final manuscript.
